# Genomic Considerations for the Modification of *Saccharomyces cerevisiae* for Biofuel and Metabolite Biosynthesis

**DOI:** 10.3390/microorganisms8030321

**Published:** 2020-02-26

**Authors:** James T. Arnone

**Affiliations:** Department of Biology, William Paterson University, Wayne, NJ 07470, USA; arnonej@wpunj.edu; Tel.: +973-720-3457

**Keywords:** *Saccharomyces cerevisiae*, gene expression, genetic engineering, neighboring gene effects, adjacent gene co-regulation, spatial positioning, position effects

## Abstract

The growing global population and developing world has put a strain on non-renewable natural resources, such as fuels. The shift to renewable sources will, thus, help meet demands, often through the modification of existing biosynthetic pathways or the introduction of novel pathways into non-native species. There are several useful biosynthetic pathways endogenous to organisms that are not conducive for the scale-up necessary for industrial use. The use of genetic and synthetic biological approaches to engineer these pathways in non-native organisms can help ameliorate these challenges. The budding yeast *Saccharomyces cerevisiae* offers several advantages for genetic engineering for this purpose due to its widespread use as a model system studied by many researchers. The focus of this review is to present a primer on understanding genomic considerations prior to genetic modification and manipulation of *S. cerevisiae*. The choice of a site for genetic manipulation can have broad implications on transcription throughout a region and this review will present the current understanding of position effects on transcription.

## 1. Background and Introduction

As the global population grows and countries continue to develop, the demand for fuel continues to rise. Traditional fuel sources are finite and contribute to climate change, stressing the importance of renewable sources of energy. This shift offers many benefits, including reductions in greenhouse gasses and improved energy security. Many countries use field crops such as corn, wheat, barley, sugar cane, and cassava to produce ethanol; however, their use as a biomass source competes with their use as food crops, increasing demands for land and water resources [1,2]. Although this strain is of a lesser concern on a global level, it can have substantial regional impacts, pushing for the exploration of other viable options [1].

The native metabolic pathways in diverse microbes, including wild yeasts and bacteria, are particularly attractive for use in the production of biofuels via fermentation, which can generate bioethanol from a variety of non-food crop substrates, such as glycerol [3,4]. The metabolic flexibility of fungi allows for the generation of ethanol from materials (including olive mill wastewater) and the production of microbial enzymes and lipids from milling and confectioners’ wastes [3,5,6]. Screening of diverse fungal isolates has proven effective in expanding the available repertoire of biofuels and metabolites produced from myriad starting materials [7]. The ability to reconstitute these pathways into an easier-to-culture, non-native host can increase the yield and efficiency of metabolite production. Advances in genetic engineering enhance the production of biofuels (and other metabolites), allowing synthesis in non-native species and manipulations to maximize the desired output.

A number of microbes, including *Escherichia coli, Clostridium acetobutylicum, Streptomyces venezuelae,* and *Saccharomyces cerevisiae* have proven successful in the introduction of non-native pathways in each organism [8,9,10]. Each organism has its own advantages (and disadvantages); however, the budding yeast, *S. cerevisiae,* is particularly amenable to genetic manipulation in the production of many metabolites. The use of *S. cerevisiae* as a model system is widespread and has the metabolic flexibility to ensure myriad biofuel outputs, including the production of fatty acid ethyl esters, butanol isomers, fatty alkanes, and fatty and higher alcohols [11,12]. Additionally, *S. cerevisiae* has a genome that can easily be manipulated, which is especially important, as optimization for the production of a particular biofuel output can require multiple genetic changes to eliminate competing pathways and maximize synthetic and native pathways to maximize output [13,14,15]. As a case in point, the production of isobutanol is optimized by the concurrent deletion of alcohol dehydrogenase and pyruvate dehydrogenase genes and the introduction of transhydrogenase-like shunts [16].

As *S. cerevisiae* has been extensively utilized as a model organism, myriad genetic tools have been developed and optimized for genetic manipulation; they include homologous recombination mediated in vivo mutagenesis, DNA assembly cloning to construct biochemical pathways, sequence and ligation-independent cloning, Gibson assembly cloning, and CRISPR-Cas9 genetic editing [17,18,19,20,21,22,23]. This yeast is highly versatile, with stable haploid and diploid life cycles, and can tolerate multiple simultaneous genetic changes [11]. Recent advances in CRISPR technology has allowed for the simultaneous introduction of up to six genetic modifications in a single transformation step [24]. *S. cerevisiae* can also be modified for the utilization of non-traditional nutrient sources, including cyanamide and phosphite for nitrogen and phosphorus, respectively, which can prevent the growth of contaminating microbes [25]. This combination of available cloning techniques, combined with the tolerance for significant genetic manipulation, makes this microorganism ideal for the introduction and optimization of non-native pathways for use in biofuel and metabolite production. 

Complete harnessing of the potential of *S. cerevisiae* requires a thorough understanding of the many mechanisms that are available to optimize the desired metabolite output. The use of plasmids for genetic engineering purposes, while easier, is much less stable than chromosomal manipulations, as plasmids require consistent selection for maintenance [26,27]. Chromosomal manipulations are much more stable, though there can be unintended second order effects that alter local transcription and can lead to unexpected complications. There are excellent reviews and resources that allow for a thorough understanding of gene expression and regulation in *S. cerevisiae* via *cis* regulatory DNA sequences and transcription factor interactions, global nucleosome positions, mRNA transcript lengths and signals, mRNA stability, decay, and their consequences on cell cycle, protein stability, and turnover [28,29,30,31,32,33,34,35]. The focus of this review is to provide an understanding of position effects and spatial positioning, the physical arrangement of genes along the chromosome in *S. cerevisiae,* and potential complications that can arise during chromosomal manipulations introduced by genetic engineering.

## 2. Heterochromatic Genomic Regions Silence Transcriptional Activity

Heterochromatin forms in transcriptionally repressed, typically gene poor regions of the genome. *S. cerevisiae* maintains heterochromatic regions at the telomere and sub-telomeric regions, the silent mating loci (*HML* and *HMR*) on chromosome III, and within the rDNA repeats found on chromosome XII [36,37,38]. 

### 2.1. Telomeric Heterochromatin

The very distal tips of eukaryotic chromosomes are telomeres, specialized genomic regions maintained in unique structures to prevent inappropriate homologous recombination or triggering of the DNA damage response pathways [39]. Telomeres consist of simple, non-coding repeats (the sequence is C_1-3_A/TG_1-3_) that stretch for approximately 300 base-pairs +/− 75 base-pairs per chromosome [40]. Adjacent to these telomeric repeats are telomere associated sequences (TAS), which fall into two classes: X and Y’. X TAS sequences are found on all telomeres and Y’ TAS sequences are found on approximately half of the telomeres, which can vary by strain. There is significant diversity seen within each TAS class—in terms of size, composition, and insertions and deletions that can vary significantly from telomere to telomere [40]. 

Initial characterization has focused on individual telomeres, with an emerging consensus—telomeres exclude nucleosomes and are maintained in a transcriptionally silent, heterochromatic state. The integration of a transgene into telomeric regions in *S. cerevisiae* results in transcriptional repression of the transgene and is commonly known as the ‘telomere position effect’ (TPE) [38]. Evidence for this silencing focuses on the repression of *URA3* integrated into telomere *TEL07L*, which is mediated by the spread of heterochromatin nucleated at the telomere across the transgene by the Sirtuin family proteins and Rap1p [41,42]. The ubiquitous nature of the TPE has recently come into question, as global analysis utilizing the more sensitive RNA-sequencing methods identified active transcription at many native telomeres, albeit at levels that are lower than integration sites further from the telomere. Furthermore, this study shows that while the Sirtuin proteins are localized throughout the telomeres, their activity is not the primary mechanism of transcriptional repression, as only 6% of subtelomeric genes are silenced by Sir protein activity [43]. The current understanding of transcription within these genomic regions is more nuanced—under certain growth conditions (e.g., the induction of the environmental stress response, or ESR), there is transcriptional activation of telomeric genes [44]. This last point is germane, as the culturing conditions required for biofuel and metabolite production can trigger the budding yeast’s ESR, ultimately activating transcription within these regions. Indeed, our observations found that members of the stress-induced toxin-response gene family cluster in telomeric and sub-telomeric regions, playing a role in coordinating the transcription of this regulon [45,46].

### 2.2. Ribosomal DNA and the Silent Mating Loci

In addition to the heterochromatin that forms on telomeric regions, there are two additional sites where constitutive heterochromatin forms, at the silent mating loci, called *HML* and *HMR*, found on chromosome III, and at the rDNA repeats on chromosome XII. While there are similarities between the heterochromatin found in each region, there are also important differences compared to telomeric chromatin. Many of the chromatin modification proteins required for heterochromatin formation and maintenance are shared at all three heterochromatin loci, and each exhibit a lack of post-translational modifications to the tails [47,48]. One major difference is that transcriptional silencing at both *HML* and *HMR* is stronger but only extends over a shorter distance than that at the telomeres. Silencing at *HML* and *HMR* is nucleated by *cis* regulatory elements that flank each locus (*HML-E* and *HML-I* and *HMR-E* and *HMR-I*, respectively); however, the presence of insulators limit the genomic distance of silencing [49]. The rDNA repeats sequester to the nucleolus, and the number of active versus repressed regions varies, matched to the cell cycle and the demand for ribosomes. The transcriptional activation during stress does not affect the *HML* mating locus or the rDNA repeats [44].

The formation of heterochromatin facilitates higher order chromosome structures and the maintenance of subnuclear organization. Telomeres terminate into loops that fold back upon themselves, clustering together on the nuclear periphery into distinct puncta, as seen in the Rabl nucleus [40,48,50]. The silencers flanking *HMR* form a local loop that facilitates transcriptional repression, while *HMR* and *HML* form an extended loop whereby Chromosome III folds across itself forming a long range interaction—all together, these interactions facilitate the characteristic subnuclear arrangement necessary for proper gene expression and genomic integrity [48,51].

## 3. Global Position Effects Result in Large Differences in Reporter and Transgene Expression

*Saccharomyces cerevisiae* was the first eukaryotic organism with a fully sequenced genome [52]. While estimates vary, there is consensus that there are just under 6000 protein coding genes, encoded by approximately 12 megabases of DNA divided into 16 chromosomes [52,53]. The completion of the genome sequence allowed researchers to construct the ‘Yeast Deletion Library’ (also known as the ‘Yeast Knock Out Collection’, or YKO collection), a series of isogenic yeast strains, each with a single non-essential gene deleted [54,55]. The library consists of 5,916 isogenic yeast strains with the kanamycin resistance (*KAN^R^*) gene replacing an individual open reading frame [55]. This resource allowed for rapid advances in the understanding of gene function on a level as never before, and led to further development of additional library collections, including the green-fluorescent protein (GFP) and tandem-affinity purification (TAP) tagged resources [56,57].

### 3.1. The Neighboring Gene Effect

The YKO collection expanded the understanding of gene function, significantly increasing the number of annotated gene functions. The design of the YKO collection integrated a *KAN^R^* under the regulation of a relatively high strength promoter. Systematic characterization of genetic screens that used the deletion library led to a surprising result: the *KAN^R^* integration site frequently disrupted the expression of a neighboring, adjacent gene [56,57,58,59]. The disruption caused by *KAN^R^* to the expression of the adjacent gene was coined the ‘neighboring gene effect’ (NGE), which is estimated to affect transcription at 7%–15% of the targeted loci [58]. Follow-up analysis revealed that this effect results in miss-annotation of genetic interactions in up to 10% of screen results, rather than the identification of a bona fide genetic link (Table 1) [59]. 

### 3.2. Global Position Effect Variance

The completion of the sequencing of the yeast genome allowed for further characterization of transcriptional differences that arise when other, identical constructs are integrated into different genomic regions. These studies characterized the differences in expression of an insertion of a construct directly, as opposed to the disruption of the transcription of neighboring genes. Two studies investigated the integration of a green fluorescent protein (GFP) reporter and a red fluorescent reporter (RFP) throughout the genome and monitored position effects on their expression. When a GFP reporter was integrated at 482 genomic sites, the noise in the transcription of this reporter varied by 20-fold, resulting in a 15-fold difference at the level of protein production [60]. A global survey characterized the position effects observed when the red fluorescent protein (RFP) gene was integrated at 1044 different genomic positions. Based on location, there was a 13-fold difference in fluorescence seen between difference loci [61]. Additional analysis of *KAN^R^* expression across the yeast deletion library revealed significant differences in the level of *KAN^R^* expression from locus to locus, with upto 35% of expressional differences attributed to position effects (Table 1) [62]. The authors note that insertion of the *KAN^R^* cassette does not disrupt the local chromatin environment [62].

## 4. Adjacent Gene Co-Regulation and Functional Clustering 

### 4.1. Adjacent Gene Co-Regulation

The production of mature, translationally competent ribosomes requires the coordinated gene expression of two distinct families of protein coding genes: the 129 ribosomal proteins (RPs) and the more than 200 rRNA and ribosome biosynthesis genes (*RRB* or *Ribi*), consuming significant amounts of intracellular energy stores and under tight regulation, predominantly at the level of transcription [65,66]. Each gene family is distinct—the RPs assemble and are incorporated into the ribosome during synthesis and remain there throughout the ribosomes’ life, while the RRBs facilitate the synthesis of the ribosome but do not remain associated after the ribosome is mature and translationally competent. The different roles result in different levels of expression for each gene family. Each gene family is tightly co-regulated, enriched for distinct promoter sequences, and bind to separate, specific transcription factors [67,68,69,70]. Characterization of the genomic distribution of both families revealed that each family is found in a non-random distribution, predominantly clustered in pairs with other members of the same family [67,71].

**Figure 1:**
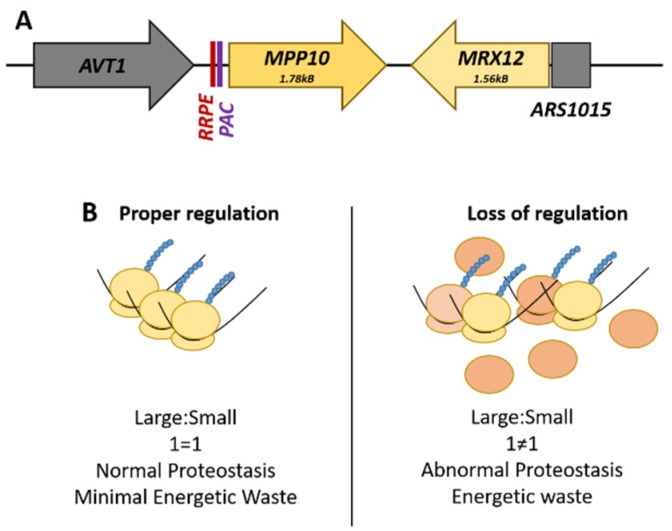
The *MPP10-MRX12* locus on Chromosome X. (**A**) The spatial arrangement of the ribosome biogenesis gene pair, *MPP10-MRX12*, highlighting the promoter regulatory elements. (**B**) Comparison of the potential effects that can arise from a loss of the proper transcriptional regulation of this gene pair. The loss of stoichiometric levels of these genes necessary for ribosome production is wasteful and could potentially alter cellular proteostasis.

The best-characterized locus is the RRB gene pair, *MPP10-MRX12*, found on Chromosome X. *MPP10*, a component of the small subunit processome and 90S preribosome complex, and *MRX12*, a protein that associates with mitochondrial ribosome, are clustered together under the transcriptional co-regulation of shared promoter elements only found upstream of *MPP10* (Figure 1A). These *cis*-regulatory promoter elements are called the ribosomal RNA processing element (RRPE) and the polymerase A and C (PAC) element, both of which are enriched within the RRB family as a whole [67]. The promoter region of *MRX12* contains no readily identifiable transcription factor-binding site; rather, the genome region immediately upstream is a nucleosome devoid autonomously replicating sequence (Figure 1) [72]. 

Functional dissection of the RRPE and PAC promoter elements in *MPP10* by mutational analysis and gene expression profiling found that these sites are necessary for the transcriptional co-regulation of this RRB gene pair with the rest of the regulon during cellular growth and the induction of the stress response [71]. There is a physical requirement of adjacency for *MRX12* to *MPP10* for their co-regulation, as insertion of an actively transcribed *LEU2* gene effectively uncouples the pair from each other; this phenomenon is termed ‘adjacent gene co-regulation’ [73]. This surprising relationship demonstrates the distance that endogenous yeast promoters can potentially act with—as transcription of *MRX12* initiates across a chromosomal distance of almost four kilobases. The advantage of this arrangement is that it can potentially help buffer the expression of components needed at roughly stoichiometric levels. The inability to do this is wasteful, potentially resulting in alterations to pools of ribosomal subunits, and ultimately to abnormal proteostasis (Figure 1B). 

### 4.2. Functional Clustering

A systematic survey in budding yeast revealed that there are many functionally related gene families found in a non-random genomic distribution—this phenomenon is not limited to genes involved in the synthesis of the ribosome. Approximately, one-quarter of functionally related gene families in *S. cerevisiae* exhibit clustering throughout the genome. This arrangement results in tighter transcriptional co-regulation throughout the cell cycle, compared to their singleton (unpaired) counterparts [74]. It is likely that there are many different drivers that lead to functional clustering in such widely diverse gene families; however, the functionally clustered genes aggregate in areas of the genome where neighboring genes influence transcription of their neighbors [45]. 

Proper gene regulation via genomic clustering can also involve transcriptional interference, which results in an anti-correlated expression of neighboring genes due to mutually exclusive expression. The canonical example of this type of transcriptional regulation is seen at the *SRG1-SER*3 genomic locus found on chromosome V. *SRG1* is a non-coding RNA that prevents the transcription of *SER3*—which can only be actively transcribed when the *SRG1* transcript is repressed—as the transcript extends over the transcription start site for *SER3*, altering the underlying chromatin [75,76]. 

**Figure 2:**
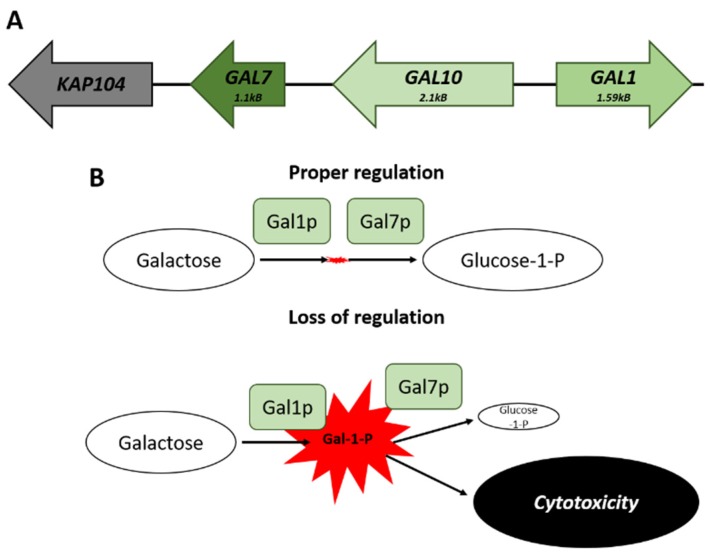
The *GAL* locus on Chromosome II. (**A**) Spatial arrangement of the genes that comprise the *GAL* locus. (**B**) Comparison of the potential effects that can arise from a loss of proper transcriptional regulation of the cluster. The failure to maintain appropriate levels of *GAL7* matched to *GAL1* leads to a buildup of Galactose-1-Phosphate and subsequent cytotoxic effects.

The co-regulation of clustered genes can help to prevent the accumulation of molecules that may be potentially toxic [74,77]. The *GAL* genes cluster on chromosome II—there are three genes necessary for galactose catabolism (Figure 2A). This locus consists of *GAL1*, a galactokinase that catalyzes the conversion of alpha-_D_-galactose to galactose-1-phosphate; *GAL10*, a UDP-glucose-4-epimerase; and *GAL7*, a galactose-1-phosphate uridyl transferase. The genomic arrangement of these genes results in buffering of the cell from transcriptional noise, maintaining comparable levels of expression, which would not be possible if the genes were unlinked. The balance in the expression of *GAL1* to that of *GAL7* avoids the accumulation of the cytotoxic galactose-1-phosphate metabolite (Figure 2B) [78].

## 5. Lessons and Conclusions

*Saccharomyces cerevisiae* offers significant advantages for the introduction of non-native biological pathways, compared to some of its less-complex, prokaryotic peers. It also presents additional levels of complexity and challenges that require consideration and planning to ameliorate. The genomic site for modification must be chosen with care, as there is the ability to introduce unintended consequences—the second order effects. The targeted integration site can profoundly affect the levels of expression of any gene—and vice versa—potentially altering delicate cellular processes and leading to a lower metabolic output. 

Global analyses of position effects reveal that centromeric and telomeric regions, a distance defined as +/− 20 kB from the repeat sequence, result in reduced gene expression compared to integration sites positioned further away [61]. These regions, however, are not completely silent in all cases. The researcher looking for a site to target for lower levels of gene expression may very well consider these regions. Genomic regions further away represent target sites that are more conducive for higher levels of gene expression, although position effects only result in high levels of expression in 25% of the genome. Hot spots that support high levels of expression include the *YDR448W*, *YGR240C*, *YHR142W*, *YML059C*, *YPL014W*, and *YPR028W* loci, and represent excellent starting points for researchers who want to synthesize highly expressed pathways [61]. One caveat is the effect of any non-native metabolite on the budding yeast. *S. cerevisiae* has a robust stress response, which triggers easily [46,79]. Such a response could result in the increase in transcription of regions that are typically low expressed and compound matters.

Our research group previously performed an analysis of the position effects on endogenous gene expression that arose due to the budding yeast’s ESR [45]. Analysis focused on the induction of the environmental stress response, including a heat-shock response, DNA damage response, oxidative stress, nitrogen depletion, and the switch in carbon source from glucose to glycerol. As a resource for those concerned about induction of the ESR, the data from 30 representative loci have been parsed from that dataset and is presented in Table 2.

Due to its versatility and potential, *S. cerevisiae* will continue to be an attractive choice for genetic engineering for a variety of purposes, both inside and outside the biofuel sector. Yeasts are important sources of metabolites within the pharmaceutical and biotechnology industries, with S. *cerevisiae* widely used for these purposes, including for the production of metabolites such as dihydroartemisinic acid (an important precursor molecule in the production of the anti-malarial agent artemisinin), and the production of beta-carotene [80,81,82,83]. Years of research and discovery on the microorganism has opened up incredible avenues and opportunities to harness this biological system as a mini factory. Furthermore, these observations are not simply limited to *S. cerevisiae*; they likely extend throughout the fungal kingdom and to related eukaryotes [74,84].

## Figures and Tables

**Table 1:** Summary of Position Effects on Gene Expression Characterized in *S. cerevisiae*.

Construct/Analysis	Position Effect Observed	Ref.
*pTEF1-LacZ* and *pACT1-LacZ*	8-fold difference in expression across 20 integration sites.	[63]
*pPRS3-LacZ*	14-fold difference in expression across 18 integration sites.	[64]
*pTEF-KAN^R^*	4-fold variation in expression across chromosome I.	[62]
*pTEF-KAN^R^* in yeast knock out collect	Characterized the ‘Neighboring Gene Effect’. Transcription disruption by the KAN^R^ cassette resulted in missannotation of gene function in genetic screens (parenthesis represents frequence of missannotation): telomere length mutant screen (24.1%), resoponse to rapamycin screen (21.5%), topoisomerase mutant sensitivity (15.7%), and 5-fluorouracil sensitivity (7.2%).	[58]
Double deletion mutant screen	‘Neighboring Gene Effect’ resulted in 18% global missannotation of a systematic synthetic double deletion screen.	[59]
*pRPL5-GFP*	Characterized effects from 63 loci on chromosome I and 482 total sites globally. 22.6% of integration sites exhibited significant expressional differences, 36.5% demonstrated significant transcriptional noise, and there was 15-fold deviation in overal expression levels.	[60]
*pTEF1-RFP* and *pURA3-RFP*	13-fold difference in expression across 1044 integration sites. Different promoters resulted in different overall levels of expression; however, position affected both constructs to the same degree.	[61]

**Table 2:** The Spearman’s correlation coefficient for 30 genomic regions during stress response and nutrient stressors.

Chromosome	Arm	Coordinates	SCC (10kB Window) *	SCC (10 Gene Window) **
II	R	415,500	0.0340	0.0130
IV	R	1301,500	−0.0500	−0.0280
IV	R	1451,200	−0.0160	−0.0112
IV	L	130,100	−0.0170	0.0030
IV	L	117,200	−0.0140	−0.0220
IV	L	217,200	0.0100	0.0330
IV	L	308,200	0.0240	0.0350
V	L	53,400	−0.0404	−0.0250
VII	L	254,000	−0.1820	−0.0750
VII	L	366,200	−0.0720	−0.0200
VII	R	649,200	−0.0390	−0.0220
VII	L	310,400	−0.0320	−0.0330
VII	R	270,600	0.0330	−0.0083
VIII	L	36,200	0.0690	0.0480
IX	L	316,400	−0.3000	0.0850
IX	L	99,600	0.0600	−0.0043
X	L	75,600	0.0644	0.1030
XI	L	431,500	0.0011	−0.0080
XII	R	932,000	−0.0500	0.0120
XII	R	805,500	−0.0190	0.0100
XII	R	282,600	0.0380	0.0620
XII	R	522,600	0.0720	−0.0280
XII	R	1028,500	0.3100	0.2470
XIII	R	754,500	0.0900	0.0300
XIV	L	330,800	−0.0470	−0.0680
XIV	L	65,200	−0.0190	−0.0300
XV	R	444,200	−0.0038	0.0211
XV	L	80,000	0.0053	−0.0610
XVI	L	173,000	0.0180	0.0290
XVI	L	135,600	0.1040	0.1170

* The average SCC for every pairwise combination of genes throughout a kB window centered on the specified target region. ** The average SCC for every pairwise combination of genes throughout a 10-gene window centered on the specified target region (25kB–30kB region).
